# Scimitar syndrome in a four-month-old infant

**DOI:** 10.4314/gmj.v57i4.9

**Published:** 2023-12

**Authors:** Frank Owusu-Sekyere, Victoria M Adabayeri, Efua Otoo, Claudia Adja-Sai, Akosua M Boateng

**Affiliations:** 1 Department of Child Health, Korle Bu Teaching Hospital, Accra, Ghana; 2 Department of Pathology, Korle Bu Teaching Hospital, Accra Ghana

**Keywords:** Scimitar syndrome, venolobar, infant, Ghana

## Abstract

The Scimitar syndrome or pulmonary venolobar syndrome is a rare, complex and variable congenital anomaly of cardiopulmonary development characterised by an abnormal right-sided pulmonary venous drainage in the inferior vena cava, malformation of the right lung, abnormal arterial supply and sometimes cardiac malformations. These serious anomalies notwithstanding, their presentation is varied, ranging from asymptomatic to severe symptoms, particularly in the neonatal and early infantile period. Some symptomatic cases are misdiagnosed as recurrent lower respiratory tract infections or as heart failure, missing the underlying congenital anomaly. Considering associated serious complications such as pulmonary hypertension and a high mortality rate, accurate and timely diagnosis is mandatory. This requires a high index of suspicion, not only by clinicians but also by radiologists who encounter suggestive chest radiographs in asymptomatic children. Still, the classic radiological finding for which the condition is named is seen in only about 50% of all cases and only 10% in affected infants. We highlight this case to heighten clinicians' and radiologists' suspicions about Scimitar syndrome.

**Funding:**

None declared

## Introduction

Scimitar syndrome is named after its radiological appearance of an anomalous vessel appearing like the classic curved Turkish sword known as a “*scimitar*.” It was first described by Cooper in 1836 at an autopsy of an infant in London^1^, and in 1960 Neill *et al.* coined the term scimitar.^2^ The first diagnosis in a live patient was made by Dotter *et al.* using cardiac catheterisation.^3^ The incidence is reported as 1-3 per 100,000 in the general population and 0.6 per 1000 in children with congenital heart disease.^4^,^5^ Female to male ratio is 2:1.^6^

The exact cause is unknown, but familial occurrence has been described with an autosomal dominant pattern with variable penetrance.^2^ It originates from a basic developmental disorder of the developing lung bud during early embryogenesis. The migratory lung bud initially receives its blood supply via a plexus from the post-branchial descending aorta. Later in development, the blood supply switches to one from the sixth aortic arch, which later becomes the pulmonary artery. Failure of this switch results in a situation where a branch of the abdominal aorta supplies the right lung. This results in the underdevelopment of the right pulmonary artery and right lung.^4^

A diagnosis of Scimitar syndrome involves demonstrating a partial anomalous pulmonary venous return to the inferior vena cava, the inferior cavo-atrial junction or the lower part of the right atrium. This vein, described as the Scimitar Vein (SV), is usually a single vein that runs anteriorly to the hilum of the lung and pierces the diaphragm to join the inferior vena cava.^4^ In addition to the SV, Scimitar syndrome is associated with abnormal right lung lobation, right lung hypoplasia, and dextroposition of the heart - the so-called triad of Scimitar syndrome.^4^,^8^ Other abnormalities infrequently seen in Scimitar syndrome are hypoplasia of the right pulmonary artery, systemic arterial blood supply to the right lower lung from the infradiaphragmatic aorta, atrial septal defect (ASD) of the secundum type and right-sided-diaphragmatic hernia.^4^,^9^

The infantile Scimitar syndrome has a high incidence of ASD and other cardiovascular abnormalities, including aortic arch abnormalities, ventricular septal defect, patent ductus arteriosus, tetralogy of Fallot, anomalous origin of the left coronary artery and truncus arteriosus.^4^

Manifestation of this syndrome is variable depending on the underlying defect and age at presentation, but clearly bimodal. There are infantile and paediatric/adult forms of presentation. The infantile form is diagnosed within the first few months of life with tachypnoea, heart failure and failure to thrive from the massive right to left shunt from the scimitar vein, associated cardiac lesions and consequent pulmonary hypertension. The presentation of the paediatric/adult form is less dramatic, usually asymptomatic and diagnosed incidentally on chest radiograph due to low right to left shunt and milder pulmonary hypoplasia.^10^,^11^ We present a case report of a four-month-old female who was diagnosed with this condition post-mortem. Verbal informed consent was obtained from the parents of this child for publication.

## Case Report

A four-month-old female was referred from a peripheral health facility to the Child Health Emergency Room of the Korle Bu Teaching Hospital (KBTH), Accra, Ghana, with acute respiratory distress secondary to a suspected mass in the chest on an x-ray.

The child presented with a six-day history of difficulty in breathing and a five-day history of intermittent fever. Her parents started her on an over-the-counter antipyretic. She was sent to the referral facility four days later based on worsening tachypnoea, where she was admitted for 2 days and treated for pneumonia with intravenous cefuroxime. She was referred based on an x-ray suspicion of a chest mass. There was a history of fast breathing and sweating during breastfeeding from birth. Weight gain had been inadequate, according to parents.

Pregnancy, including antenatal anomaly scans and delivery history, was unremarkable. There was no history of maternal ingestion of alcohol, illicit drugs or herbal preparations in pregnancy. Immunisation was up to date for her age. Developmental milestones were appropriate for age, and the child was exclusively breastfeeding. There was a positive maternal family history of asthma but no other chronic illnesses. Mother was diagnosed with syphilis, but no treatment was given.

Initial examination revealed a very ill infant in severe respiratory distress. The baby was moderately pale, not cyanosed, anicteric, well hydrated with a temperature of 37.9°C, and had no obvious dysmorphic facies. Her weight of 4.4kg was below the third centile for age. Her birth weight was 3.1kg. Head circumference was 38cm (below the third centile), and oxygen saturation was 82% on room air, which improved to 93% on oxygen at 5L/min. Upon cardiovascular examination, the heart rate was 164 bpm. Peripheral pulses, including femorals, were of good volume and synchronous.

The heart sounds were pronounced in the right hemithorax, and an ejection systolic murmur was present at the pulmonary area. She was tachypnoeic (respiratory rate of 80/min) with flaring of alae nasi and intercostal and subcostal recessions. Air entry was reduced bilaterally, both anterior and posteriorly, with bronchovesicular breath sounds bilaterally. Crepitations were heard bilaterally but more on the left hemithorax with inspiratory rhonchi. There was hepatomegaly of 5cm below the costal margin and splenomegaly 4cm. Examination of the central nervous system was normal. The baby was initially managed for right lobar pneumonia with dextrocardia and congestive cardiac failure. Oxygen, Ceftriaxone and Furosemide were initiated.

**Figure 1:**
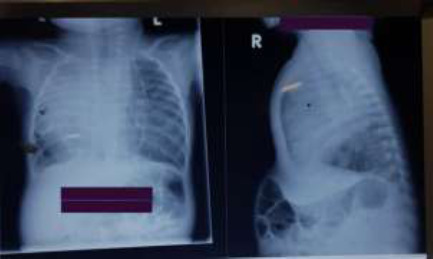
Shows dextroposition with mediastinum shift to the right, significant reduction of right lung volume, obliteration of right heart silhouette and right hilum and consolidation of the right upper and middle lung lobes with bronchial wall thickening and interstitial shadowing on the left

The haemoglobin was 9.6g/dl with neutrophilia of 24.19 x10^9^L. Blood urea and electrolytes (sodium, potassium and chloride) were normal. A plain chest radiograph (Figure 1) showed dextroposition with normal visceral situs, right upper and middle lobe consolidation, reduced right lung volume and bronchial wall thickening with interstitial shadowing on the left. The baby was very agitated with clinical evidence of impending respiratory arrest; hence, only a focused echocardiogram could be done. The echocardiogram showed cardiac dextroposition and hypertrophy of the right ventricle.

In view of worsening respiratory distress, the baby was admitted to the Paediatric Intensive Care Unit (PICU) with oxygen saturations ranging between 87% - 90% on oxygen via nasal prongs. Saturations improved to 100% when the child was put on bubble continuous positive airway pressure (CPAP) with a positive end-expiration pressure of 4cmH20 and flow rate of 8L/min. She maintained oxygen saturations on CPAP for about 24 hours but later began to desaturate, necessitating intubation and mechanical ventilation. Due to persistent pyrexia above 39.0°C, ceftriaxone was empirically upgraded to Meropenem after a blood culture sampling. No bacteria was isolated. Even on the Mechanical ventilator, she struggled to maintain adequate oxygen saturation with a maximum of 88% on 100% oxygen. She suffered a cardiac arrest on the third day of mechanical ventilation and died.

**Figure 2:**
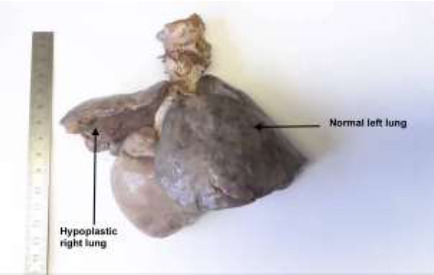
a firm left lung and hypoplastic right lung

**Figure 3:**
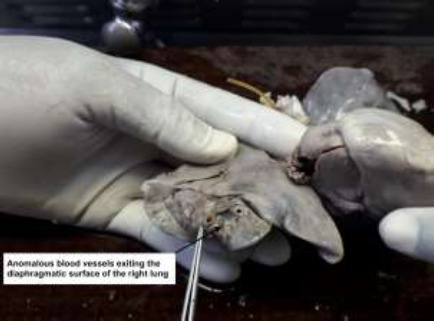
Partial anomalous pulmonary venous drainage

**Figure 4:**
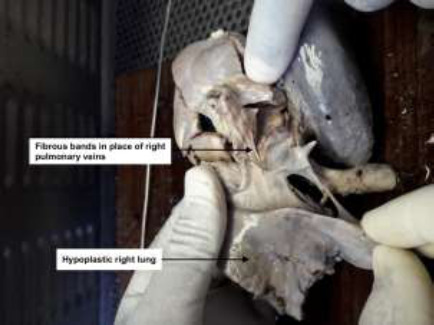
Fibrous bands representing atretic right pulmonary veins

A post-mortem revealed a firm left lung and hypoplastic right lung (Figure 2), partial anomalous pulmonary venous drainage (Figure 3), fibrous bands representing atretic right pulmonary veins (Fig 4), hypoplastic left atrium and ventricle and hypertrophic right atrium and ventricle. The post-mortem diagnosis was diffuse alveolar damage as the immediate cause of death with congenital pulmonary venolobar (Scimitar) syndrome.

## Discussion

Congenital venolobar (Scimitar) syndrome (also called hypogenetic lung syndrome) is a rare variant of partial anomalous pulmonary venous return with an aberrant vein.^13^ It is inherited via the autosomal dominant pattern with variable penetrance.^14^ There was no family history in our case. Although the syndrome has been associated with dysmorphic features^15^, our patient had no obvious dysmorphic features. Symptomatic presentation, although variable, could be immediately after birth with a median age of presentation as seven months for the infantile type. Many patients, however, remain asymptomatic throughout childhood presenting only with recurrent respiratory tract infections. Pulmonary arterial hypertension has been recognised as the underlying pathology for severe symptoms with poor prognosis.^10^,^14^ Our patient had 2 out of 4 poor prognostic factors namely, stenosis of the anomalous pulmonary veins, presence of systemic arterial supply to the right lung, reduction of the pulmonary vascular bed on the right side, and increased pulmonary blood flow from anomalous drainage or the presence of intracardiac lesion.^10^ Interestingly, there was no intracardiac anomaly despite the early symptomatic presentation of our case.

The present case highlights the diagnostic difficulty of Scimitar syndrome. The reduced right lung volume, ipsi-lateral mediastinal shift and dextroposition of the heart were referred to as a chest mass but should have raised suspicion of pulmonary hypoplasia. However, the pathognomonic feature of an SV on the chest radiograph was not apparent in our case, which is in keeping with reports that the typical scimitar radiographic finding is only present in approximately half of all patients and less than 10% with the infantile type.^4^ An echocardiogram, the investigation of choice, is good at delineating the scimitar vein, cardiac anomalies and any arterial supply to the right lung. However, it has difficulties defining small or absent anomalous venous drainage in infants.^16^ A detailed study could not be done because of her unstable status. A foetal echocardiogram is a useful prenatal screening method in highlighting obstructed pulmonary venous pathways and the confluent veins.^17^ The standard four-chamber antenatal cardiac scan was normal in her case. Unfortunately, a detailed foetal echocardiogram, necessary for antenatal diagnosis, is not available in most centres in Ghana. An electrocardiogram usually shows right axis deviation and right ventricular hypertrophy. A 3-dimensional computerised tomography angiogram or magnetic resonance angiogram would have provided excellent structural detail of the heart and vascular connections. 17,18 Unfortunately, these scans are not easily available and affordable countrywide. There is the need then to strengthen the capacity to offer these services countrywide. In our case, though, the scan was not done because transporting the child for the scans was risky.

Because of the varied clinical spectrum for scimitar syndrome, medical management depends on the severity of presentation and the amount of blood flowing to the inferior vena cava from the anomalous pulmonary veins. Medical intervention is indicated in those with smaller drainage. Such patients often respond to supportive care and diuretics for heart failure. Antibiotic administration for chest infections, good nutrition, oxygen supplementation and ventilatory support are additional recommended management options, which were all utilised in our patient.^19^

Surgical treatment of Scimitar syndrome is required in symptomatic patients, especially those with other associated cardiac abnormalities. Surgical options include redirecting the venous drainage to the left atrium, ligation or embolisation of vascular supply to the sequestered lobe and pneumonectomy.^4^ Surgical repair reestablishes normal blood flow to the right lung, abolishing the increased left-to-right shunt and its resultant pulmonary volume overload. Simple ligation or coil embolisation of abnormal arterial vessels is the best and simplest treatment, particularly in symptomatic infants as in our patient.^4^ Unfortunately, this surgical intervention, although available and affordable, could not be performed before her demise.

## Conclusion

Accurate diagnosis and management of patients with Scimitar syndrome can be challenging in resource-limited settings. Limited expertise in foetal echocardiogram, delays in referral and limited access to diagnostic investigations and interventions are frequently encountered, as illustrated in this case.

Respiratory distress and cardiac dextroposition should raise suspicion of Scimitar syndrome for prompt diagnosis and treatment.
